# Assessment of Eight Entrepreneurial Personality Dimensions: Validity Evidence of the BEPE Battery

**DOI:** 10.3389/fpsyg.2018.02352

**Published:** 2018-11-29

**Authors:** Marcelino Cuesta, Javier Suárez-Álvarez, Luis M. Lozano, Eduardo García-Cueto, José Muñiz

**Affiliations:** ^1^CIBERSAM, Department of Psychology, University of Oviedo, Oviedo, Spain; ^2^Directorate for Education and Skills, Organisation for Economic Co-operation and Development, Paris, France; ^3^Mind, Brain and Behavior Research Center, Department of Methodology of Behavioral Sciences, University of Granada, Granada, Spain

**Keywords:** personality, entrepreneurs, assessment, validity evidence, Big Five

## Abstract

**Background:** The study of entrepreneurial activity has undergone intense development in recent decades. Traditionally this topic has been addressed from three approaches: economic, sociological and psychological. In the study of enterprising personality, two fundamental perspectives stand out: the use of general personality traits, like the Big Five, and the use of more specific traits related to entrepreneurial spirit, such as self-efficacy, autonomy, innovation, optimism, and others. The objective of this study is to provide validity evidence for a new instrument for measuring eight specific dimensions of entrepreneurial personality (BEPE).

**Methods:** The sample was composed of 1,170 adults from the general population (59.9% women). The average age was 42.34 years with a standard deviation of 12.96. Of the sample, 13% were self-employed. Internal factorial structure and reliability of BEPE were examined. The relationships with other variables and the discriminative capacity of the BEPE between different groups of workers were analyzed.

**Results:** First order exploratory factor analyses show the essential unidimensionality of each of the eight proposed sub-scales, with factorial weights ranging between 0.341 and 0.825. In the Confirmatory Factor Analysis, the best fit was achieved with a Bifactor model. With regards to reliability, the eight BEPE sub-scales gave high alpha coefficient values, between 0.81 and 0.89, as did the total battery (0.97). BEPE sub-scales show a high canonical correlation with the Big Five personality factors (0.796) and with the sub-scales of the Measure of Entrepreneurial Talents and Abilities questionnaire (0.779).

**Conclusion:** The BEPE questionnaire for the evaluation of the eight fundamental specific dimensions of the entrepreneurial personality presents adequate psychometric properties. Its relationships with other measures of personality traits are in line with what is expected. Therefore, the BEPE is a new measurement instrument that can be used with confidence both in the applied field and in research.

## Introduction

Entrepreneurial activity is considered to be a crucial element in the development of a market economy (OECD/The European Commission, 2013), which is why it is regularly monitored by large international organizations in various countries (OECD, 2017; Global Entrepreneurship Monitor [GEM], 2018). Its importance has led to a substantial increase in research in recent years (Rauch and Frese, 2007a; Sánchez, 2011; Liñán and Fayolle, 2015; Suárez-Álvarez and Pedrosa, 2016; Chandra, 2018). A multidisciplinary approach has been predominant, with three main branches, economic, sociological, and psychological, the latter including cognitive, emotional, attitudinal and personality aspects. There are two main lines of investigation in research into personality characteristics of entrepreneurs, and those two lines provide the basis for this study. On the one hand, there are those who consider the Big Five type personality traits to be the appropriate paradigm for studying entrepreneurs’ personality characteristics (Zhao et al., 2010; Brandstatter, 2011), and on the other, those who propose using more specific traits linked to entrepreneurial spirit (Rauch and Frese, 2007a,b; Almeida et al., 2014; Suárez-Álvarez et al., 2014). Those who hold the former position invoke the predictive capacity of classic Big Five personality models, while those in favor of the latter advocate the use of specific traits to account for entrepreneurial personality. Empirical results suggest that specific traits would have added predictive capability compared to more general models (Leutner et al., 2014).

There is a great tradition of evaluating general personality traits and a profusion of measuring instruments for that purpose, whereas the evaluation of specific entrepreneurial personality traits is relatively recent, as are the instruments which have emerged. Notable instruments include the Entrepreneurial Aptitude Test (Favretto et al., 2003), the Skills Confidence Inventory (Betz et al., 2005), General Enterprising Tendency (Caird, 2006), the Entrepreneurial Intention Questionnaire (Liñán and Chen, 2006), the Entrepreneurial Guidance Questionnaire (Sánchez, 2010), the Measure of Entrepreneurial Talents and Abilities, META (Ahmetoglu et al., 2011), and the High Entrepreneurship. Leadership and Professionalism, HELP (Di Fabio et al., 2016). In an International context, the META questionnaire has probably been the most widely accepted (Ahmetoglu et al., 2011; Almeida et al., 2014; Leutner et al., 2014). A detailed analysis of these instruments’ characteristics can be found in Suárez-Álvarez and Pedrosa (2016). Most of these measuring instruments have focused on a specific trait in an entrepreneur’s personality, so there are no comprehensive, exhaustive, systematic analyses of entrepreneurial personality because the instruments each focus on a single important dimension. It is also surprising that their development and analysis has not made use of the advantages of Item Response Theory models (van der Linden, 2016).

To attempt to overcome these limitations, the BEPE battery was developed. Initially, the development was made for young people as this is a key period in the emergence of entrepreneurial initiatives (Damon et al., 2015; Obschanka et al., 2017). This evaluates eight dimensions of entrepreneurial personality that were identified following an exhaustive literature review. The BEPE battery for young people and adolescents exhibits good psychometric properties, both in its classic version (Muñiz et al., 2014; Suárez-Álvarez et al., 2014), and its computerized adaptive version (Pedrosa et al., 2016). The main objective of this study is to adapt the BEPE battery to an adult population and to provide evidence of validity to support its use in research and applied situations. Evidence of validity will be gathered about internal structure, the relationship to other variables, and the discriminative capacity between different groups of self-employed and employees.

## Materials and Methods

### Participants

The initial sample was made up of 1,324 volunteers from the general adult population, found by a non-random snowball procedure. The participants were contacted via email and responded to various measurement instruments on the internet. The procedure was as follows: the members of the research team contacted personally with known people who fulfilled the desired characteristics (over 18 years of age and working). These people were asked for their e-mail as well as the e-mail of other possible participants. We contacted them individually (via e-mail) asking them to collaborate in the research and to provide new e-mails from people who could participate in the research. The procedure was kept active for 3 months. The initial sample was reduced to 1,170 participants, 59.9% women, after eliminating those who exhibited insufficiently rigorous behavior when responding to the questionnaires as measured by a control of attention scale. The mean age was 42.34 years, with a standard deviation of 12.96. Of the participants who were evaluated, 13% were self-employed.

### Instruments

#### Battery for the Assessment of the Enterprising Personality (BEPE)

This is a questionnaire which evaluates the eight specific personality dimensions identified in the literature as the most promising when characterizing entrepreneurial personality: Self-efficacy, Autonomy, Innovativeness, Internal locus of control, Achievement motivation, Optimism, Stress tolerance, and Risk-taking (Baum et al., 2007; Rauch and Frese, 2007a,b; Muñiz et al., 2014; Suárez-Álvarez et al., 2014). The items making up the battery follow a Likert-type format with five answer categories (1 totally disagree, 5 totally agree), in line with established psychometric literature which indicates that between four and six answer categories produce better psychometric indicators (Lozano et al., 2008).

Each of the scales is briefly described below; for more detail on their definition and the process of construction see Suárez-Álvarez et al. (2014). *Self-efficacy* refers to a person’s conviction that they can organize and carry out actions effectively, and their persistence when they encounter obstacles to reaching their goals (Costa et al., 2013). *Autonomy* refers to the motivation for entrepreneurial creation as an attempt to achieve a certain individual freedom (Van Gelderen and Jansen, 2006). *Innovativeness* is about the will and interest in finding new ways to do things (Rauch and Frese, 2007b). *Achievement motivation* can be defined as the desire to achieve standards of excellence (Rauch and Frese, 2007b; Suárez-Álvarez et al., 2013). *Internal locus of control* is about the causal attribution of consequences of one’s own behavior (Rauch and Frese, 2007b; Chell, 2008; Suárez-Álvarez et al., 2013). *Optimism*, is defined as the beliefs a person has about good things happening more than bad things in their life (Shepperd et al., 2002). *Stress tolerance* may be defined as the resistance to perceiving environmental stimuli as stressors thanks to the appropriate use of coping strategies (Lazarus and Folkman, 1984). *Risk-taking* is people’s tendency and will to assume certain levels of risk or change to achieve an objective which offers more benefits than negative consequences (Moore and Gullone, 1996).

The original battery was designed for the evaluation of young people (Muñiz et al., 2014), and demonstrated adequate psychometric properties. The adaptation of this original version for the general adult population was carried out as follows. The 87 items in the original BEPE were reformulated to make the language suitable for an adult population, and new items were constructed aimed directly at the general adult population. Following a thorough literature review on the topic, work began with a bank of 161 items. This initial bank of items was evaluated by 15 experts in psychological assessment, who were asked to rate each item on a scale of 1 to 10 in terms of their suitability for a general population. Items scoring less than 8 were rejected or reformulated. Following this first filter, the items were given to 142 psychologists to evaluate the suitability of each item in terms of measuring the BEPE dimension it was supposed to address. Items scoring less than 9, on a scale of 1–10, were revised. Finally, a quantitative pilot study was performed with a sample of 132 participants. Discrimination indices were calculated (item-test correlation) for the items, and an exploratory factor analysis was performed for each of the eight BEPE sub-scales. After eliminating items which did not meet psychometric quality criteria (discrimination index >0.20 and factorial loadings >0.3), each sub-scale finally consisted of 15 items (Muñiz et al., 2005).

#### Measure of Entrepreneurial Tendencies and Abilities (META), (Ahmetoglu et al., 2011)

Measure of entrepreneurial tendencies and abilities (META) is a self-report scale with 44 items, which measures personality traits relevant to business success. It has four dimensions defined as follows (Ahmetoglu and Chamorro-Premuzic, 2013): *Proactivity*, the tendency to be proactive about projects and get things done (relates to energy, confidence and self-determination); Creativity, the ability to generate innovative business ideas (relates to non-conformity, originality and preference for novel experiences); *Opportunism*, the tendency to identify new business opportunities (relates to being alert, informed, and detecting future trends); and *Vision*, the ability to see the bigger picture, the motivation to bring change and create progress (relates to values and having a higher sense of purpose). The items are measured on a five-point Likert-type scale which ranges from “completely disagree” to “completely agree.” The four scales exhibit appropriate values of internal consistency (Ahmetoglu and Chamorro-Premuzic, 2013): Proactivity (0.84), Creativity (0.83), Opportunism (0.86), Vision (0.76). In our sample the alpha values were: Proactivity: 0.70, Creativity: 0.81, Opportunism: 0.86, and Vision: 0.76.

#### NEO Five Factor Inventory (NEO-FFI), (Costa and McCrae, 1985)

The Spanish version from Cordero et al. (2008) was used. This questionnaire evaluates the Big Five personality factors: Neuroticism, Extraversion, Openness, Agreeableness, and Conscientiousness. Each scale is made up of 12 Likert-type items with five response categories ranging from total disagreement, to total agreement. It exhibits good psychometric properties, with sub-scale reliability coefficients above 0.80 (Cordero et al., 2008): Neuroticism (0.90), Extraversion (0.84), Openness (0.82), Agreeableness (0.83), and Conscientiousness (0.88). The alpha reliability coefficients calculated in our sample were: Neuroticism: α = 0.88 and ω = 0.90, Extraversion: α = 0.83 and ω = 0.86, Openness: α = 0.81 and ω = 0.83, Agreeableness: α = 0.73 and ω = 0.76, and Conscientiousness: α = 0.81 and ω = 0.85.

#### Control of Attention Scale

This is a scale comprising 10 Likert-type items with 5 response categories. Its objective is to detect those participants who respond to evaluation instruments randomly or carelessly. The questions are of the type “In this question you must select the option *completely agree.*” Participants responding incorrectly to two or more items were eliminated. By this criterion, 154 participants (11.6%) were removed from the study.

### Procedure

The measuring instruments were administered via the internet in the same order for all participants. An application developed “*ad hoc*” by the research team was used for administration of the instrument. It was not considered appropriate to carry out a time control since it is a typical execution scale. However, a scale was included to control the quality of the response (see instruments section). The average response time, estimated in the test phase, to the three tests (BEPE, META, and NEOFFI) was 40 min. Participants were contacted by email and completed the aforementioned instruments anonymously in a single session.

### Data Analyses

Following the model of similar works (Chen and Lin, 2018; Sinval et al., 2018) the data analysis was carried out in different phases. Following the Standards for Educational and Psychological Testing (American Educational Research Association [AERA] et al., 2014) different procedures were used in search of validity evidences. First, the characteristics of the eight scales of the BEPE were analyzed separately using the total of the participants (1179) and the 120 items selected in the pilot study. Discrimination indices were calculated (item-test correlation), and Exploratory Factor Analyses were performed with polychoric correlation matrices. ULS (Unweighted Least Squares) was used as an extraction method, and the number of factors to retain was determined by parallel analysis (Horn, 1965), the percentage of variance explained, and the model fit indices based on study of residuals (GFI and RMSR), as they are the most suitable and independent of the method of estimation (Ferrando and Lorenzo-Seva, 2017). Model fit is considered adequate when GFI is greater than 0.09 and RMSR is less than 0.08 (Kline, 2011). The 10 items of each scale with the highest factorial load were selected.

In the second phase, the total sample was randomly divided into two subsamples. In the first subsample, of 390 participants, EFA was performed. In the second, of 780 participants, CFA models were adjusted. In the first subsample EFA of each scale was performed with the same characteristics as in the first phase and an exploratory Bifactor analysis was also carried out. Over the sample of 780 participants, three CFA models were adjusted: a model with 8 first-order factors, a model with 8 first-order factors and a second order factor and a Bifactor model with a general factor and 8 group factors WLSMV was used as estimator and Chi-square/df, RMSEA, CFI, and TLI were used as adjustment indices. It is considered that there is a good fit when Chi-square/df < 5, CFI and TLI > 0.95 and RMSEA < 0.08 (Hu and Bentler, 1999; Jackson et al., 2009). The change in CFI (ΔCFI) was also calculated. A ΔCFI larger than 0.01, between nested models, indicates a meaningful change in model fit (Wang and Wang, 2012).

To look for validity evidence Pearson correlations between the BEPE sub-scales and the META and NEO-FFI sub-scales were calculated, along with the canonical correlation between BEPE and META sub-scales, and between BEPE and NEO-FFI sub-scales in order to understand the overall relationship between the two blocks of variables. In addition, in order to estimate the common variance between the blocks of variables, the redundancy coefficient was calculated. To analyze convergent validity evidence, the average variance extracted (AVE) was estimated as described in Fornell and Larcker (1981). Values of AVE ≥ 0.5 were considered adequate (Hair et al., 2009).

Evidence of discriminant validity understood as the items representing a dimension are not strongly correlated with other dimensions was assessed by comparing the AVE of the scales with the squared correlation of the scales (Fornell and Larcker, 1981; Marôco, 2014). For two factors x and y, if AVEx and AVEy ≥*r*^2^_xy_ (Fornell–Larcker criterion) there is evidence of discriminant validity.

The capacity of BEPE to differentiate between groups was examined via Multivariate Analysis of Variance (MANOVA), linear regression and binary logistic regression.

Scale reliability was calculated via the alpha coefficient (Cronbach, 1951) and ω de McDonald (McDonald, 1999).

Data were analyzed using SPSS24 (IBM Corp, 2016) for sub-samples selection, correlations, MANOVA and regressions; EFA analyses were performed with FACTOR10.5.03 (Lorenzo-Seva and Ferrando, 2013) and Mplus8 (Muthén and Muthén, 2017) was used for CFA.

## Results

### Factor Related Validity Evidence

The analysis of the test items was performed separately for each of the eight defined scales. Using the full sample item discrimination indices were calculated (item-test correlation), all of which were above 0.20, ranging from 0.287 to 0.705.

The Exploratory Factorial Analysis gave statistically significant values of Bartlett’s Sphericity Index (*p* < 0.01) and Kaiser–Meyer–Olkin (KMO) indices above 0.85 in all cases. Factor loadings were between 0.341 and 0.825. In each of the eight scales GFI values were above 0.95 except for Internal locus of control (0.936). RMSR was below 0.08 with the exception of Locus of control (0.122) and Stress tolerance (0.094), and in all cases the percentage of variance explained by the first factor was over 30%. From these results, each scale may be considered essentially unidimensional (Kline, 2011). In order to reduce the number of items and achieve more homogeneous scales, the 10 items of each scale with the highest factor loadings were retained.

Using SPSS, the total sample was randomly divided into two subsamples (1/3 and 2/3). In the first subsample, of 390 participants, EFA was performed, separately for every reduced scale (10 items). The Exploratory Factorial Analysis gave statistically significant values of Bartlett’s Sphericity Index (*p* < 0.01) and KMO indices above 0.87 in all cases. Factor loadings were between 0.478 and 0.879. In each of the eight scales, GFI values were above 0.98, RMSR was below 0.08, and in all cases, the percentage of variance explained by the first factor was over 45%. An exploratory Bifactor model (Reise, 2012; Cheng and Zang, 2018; Reise et al., 2018) that showed an adequate fit to the data was also tested (GFI = 0.991; RMSR = 0.0283).

In the subsample of 780 participants, three models of confirmatory factor analysis were adjusted: a model with 8 first-order factors, a model with 8 first-order factors and a second order factor and a Bifactor model with a general factor and 8 group factors. As seen in Table 1, the Bifactor model is the one that presents a better fit to the data, confirming what was found in the exploratory analysis. In Table 2 the factorial structure of the Bifactor model is shown.

**Table 1:** Fit indices of AFC.

Model	Chi-square	Chi-square/df	CFI	TLI	RMSEA	ΔCFI
Second order	10535.84	3.43	0.876	0.872	0.056	–
8 first-order factors	9990.315	3.27	0.885	0.881	0.054	0.009
Bifactor	8314.2	2.77	0.912	0.907	0.048	0.027

**Table 2:** Factor loadings of Bifactor Model.

Item	Factor loading	Item	Factor loading	Item	Factor loading	Item	Factor loading
**General factor**							

SE1	0.72	IN1	0.48	AM1	0.59	ST1	0.72
SE2	0.75	IN2	0.62	AM2	0.67	ST2	0.27
SE3	0.71	IN3	0.56	AM3	0.61	ST3	0.54
SE4	0.69	IN4	0.63	AM4	0.52	ST4	0.37
SE5	0.70	IN5	0.48	AM5	0.67	ST5	0.46
SE6	0.79	IN6	0.56	AM6	0.70	ST6	0.42
SE7	0.65	IN7	0.60	AM7	0.60	ST7	0.38
SE8	0.72	IN8	0.74	AM8	0.69	ST8	0.42
SE9	0.78	IN9	0.58	AM9	0.66	ST9	0.36
SE10	0.70	IN10	0.56	AM10	0.58	ST10	0.53
AU1	0.43	IL1	0.47	OP1	0.59	RT1	0.61
AU2	0.14	IL2	0.63	OP2	0.55	RT2	0.73
AU3	0.61	IL3	0.50	OP3	0.56	RT3	0.50
AU4	0.63	IL4	0.52	OP4	0.62	RT4	0.56
AU5	0.32	IL5	0.42	OP5	0.79	RT5	0.66
AU6	0.26	IL6	0.31	OP6	0.62	RT6	0.59
AU7	0.30	IL7	0.60	OP7	0.50	RT7	0.57
AU8	0.52	IL8	0.39	OP8	0.51	RT8	0.64
AU9	0.55	IL9	0.43	OP9	0.71	RT9	0.56
AU10	0.56	IL10	0.46	OP10	0.48	RT10	0.46

**SE**	**AU**	**IN**	**IL**	**AM**	**OP**	**ST**	**RT**

**Group factors**							

-0.02	0.47	0.48	0.69	0.40	0.57	0.20	0.39
-0.28	0.61	0.49	0.40	0.37	0.56	0.47	0.28
0.54	0.18	0.30	0.61	0.34	0.39	0.41	0.55
-0.15	0.02	0.45	0.30	0.37	0.45	0.42	0.19
0.24	0.70	0.32	0.44	-0.07	0.31	0.48	0.21
-0.02	0.66	0.49	0.43	0.05	0.44	0.74	0.60
0.49	0.67	0.49	0.24	0.26	0.58	0.67	0.14
0.12	0.52	0.35	0.64	0.06	0.47	0.47	0.28
0.11	0.36	0.41	0.55	0.46	0.19	0.50	0.18
-0.26	0.21	0.41	0.66	0.58	0.61	0.23	0.50

*SE, self-efficacy; AU, autonomy; IN, innovativeness; IL, internal locus of control; AM, achievement motivation; OP, optimism; ST, stress tolerance; RT, risk-taking*.

### Convergent Validity Evidence

Average variance extracted, calculated over the full sample, was satisfactory for some dimensions but slightly low in others: Self-efficacy = 0.54, Autonomy = 0.40, Innovativeness = 0.53, Internal locus of control = 0.47, Achievement motivation = 0.50, Optimism = 0.55, Stress tolerance = 0.40, and Risk-taking = 0.48.

### Discriminant Validity Evidence

The discriminant validity was assessed by comparing the AVE of the factors with the squared correlation of the factors (Marôco, 2014). Discriminant validity evidence is obtained when the AVE for two factors is larger than the squared Pearson correlation between the two factors. As shown in Table 3, discriminant validity is reached in all cases except Self-efficacy vs. Achievement motivation, Self-efficacy vs. Risk-taking and Innovativeness vs. Risk-taking.

**Table 3:** Discriminant validity evidence of the BEPE scales.

Scales	AVE_1_	AVE_2_	*r*^2^	Scales	AVE_1_	AVE_2_	*r*^2^
SE-AU	0.54	0.40	0.28^∗^	IN-AM	0.53	0.50	0.44^∗^
SE-IN	0.54	0.53	0.46^∗^	IN-OP	0.53	0.55	0.29^∗^
SE-IL	0.54	0.47	0.34^∗^	IN-ST	0.53	0.41	0.18^∗^
SE-AM	0.54	0.50	0.61	IN-RT	0.53	0.48	0.52
SE-OP	0.54	0.55	0.52^∗^	IL-AM	0.47	0.50	0.38^∗^
SE-ST	0.54	0.41	0.37^∗^	IL-OP	0.47	0.55	0.21^∗^
SE-RT	0.54	0.48	0.53	IL-ST	0.47	0.41	0.12^∗^
AU-IN	0.40	0.53	0.25	IL-RT	0.47	0.48	0.203^∗^
AU-IL	0.40	0.47	0.16^∗^	AM-OP	0.50	0.55	0.34^∗^
AU-AM	0.40	0.50	0.31^∗^	AM-ST	0.50	0.41	0.19^∗^
AU-OP	0.40	0.55	0.14^∗^	AM-RT	0.50	0.48	0.46^∗^
AU-ST	0.40	0.41	0.09^∗^	OP-ST	0.55	0.41	0.35^∗^
AU-RT	0.40	0.48	0.24^∗^	OP-RT	0.55	0.48	0.32^∗^
IN-IL	0.53	0.47	0.16^∗^	ST-RT	0.41	0.48	0.24^∗^

*SE, self-efficacy; AU, autonomy; IN, innovativeness; IL, internal locus of control; AM, achievement motivation; OP, optimism; ST, stress tolerance; RT, risk-taking*.

*^∗^Meets the criteria set by Marôco (2014)*.

### Reliability: Internal Consistency

The values of reliability coefficients were adequate for all scales and for the Total score. (Table 4).

**Table 4:** Reliability of the BEPE questionnaire.

	Alpha	Omega
Self-efficacy	0.883	0.921
Autonomy	0.808	0.865
Innovativeness	0.878	0.918
Internal locus of control	0.848	0.898
Achievement motivation	0.862	0.907
Optimism	0.890	0.923
Stress tolerance	0.842	0.871
Risk-taking	0.866	0.900
Total	0.965	0.963

### Relationship With Other Variables

Table 5 shows the correlations between the BEPE and META scales, which ranged between 0.174 (Internal locus of control and Creativity) and 0.693 (Innovation and Creativity). The canonical correlation between the eight BEPE scales and the four META scales was 0.779, and the redundancy coefficient was 0.311, which indicates 31.1% common variance. The correlation between the total scores of the BEPE and the META is 0.692, indicating high convergence between the two measuring instruments.

**Table 5:** Pearson correlations between BEPE and META sub-scales.

	META			
BEPE	Opportunism	Proactivity	Creativity	Vision
Self-efficacy	0.469	0.418	0.491	0.564
Autonomy	0.313	0.193	0.422	0.415
Innovativeness	0.447	0.364	0.693	0.494
Internal locus of control	0.270	0.200	0.174	0.477
Achievement motivation	0.418	0.373	0.420	0.624
Optimism	0.354	0.374	0.350	0.406
Stress tolerance	0.347	0.345	0.314	0.294
Risk-taking	0.587	0.467	0.591	0.516

Table 6 shows the relationships between the eight BEPE scales and general personality traits as measured via NEO-FFI. The highest values are seen between Stress tolerance and Neuroticism (-0.738), and between Achievement motivation and Responsibility (0.618). The canonical correlation between both sets of variables is 0.796, with a redundancy coefficient of 0.287, which means that there is 28.7% common variance between the two tests.

**Table 6:** Pearson correlations between BEPE and NEO-FFI sub-scales.

	NEO-FFI			
BEPE	Agreeableness	Openness	Extraversion	Neuroticism	Conscientiousness
Self-efficacy	0.129	0.215	0.508	-0.492	0.472
Autonomy	-0.067	0.166	0.220	-0.200	0.333
Innovativeness	0.121	0.369	0.445	-0.284	0.317
Internal locus of control	0.144	0.062	0.257	-0.266	0.486
Achievement motivation	0.111	0.202	0.407	-0.343	0.618
Optimism	0.275	0.200	0.538	-0.567	0.306
Stress tolerance	0.189	0.104	0.359	-0.738	0.337
Risk-taking	0.035	0.281	0.439	-0.339	0.279

### Differences Between Groups

Two MANOVA analyses were performed in order to examine the discriminative capacity of the test between participants grouped according to their work status. Firstly, two groups were created, people who worked for others (employees; *n* = 1018), and people who worked for themselves (self-employed; *n* = 152). Statistically significant overall differences were found (*F*_8,1161_ = 4.371, *p* < 0.001, *d* = 0.35). The self-employed participants had higher scores in all BEPE scales, although that difference was only statistically significant in Autonomy (See Table 7).

**Table 7:** Differences between self-employed and employed workers in BEPE sub-scales.

	Self-Employed	Employed	*F*	*p*	*d*
	(*N* = 152)	(*N* = 1080)			
	Mean (*SD*)	Mean (*SD*)			
Self-efficacy	37.22 (5.18)	37.55 (5.05)	0.520	0.471	0.00 no effect
Autonomy	38.72 (4.35)	40.64 (4.98)	24.846	0.000	0.29 small
Innovativeness	38.24 (4.86)	38.75 (4.50)	1.458	0.228	0.06 no effect
Internal locus of control	39.37 (4.73)	39.54 (5.36)	0.157	0.692	0.00 no effect
Achievement motivation	39.18 (4.64)	39.91 (4.43)	3.389	0.066	0.05 no effect
Optimism	38.14 (5.50)	38.77 (5.27)	1.737	0.188	0.06 no effect
Stress tolerance	32.46 (6.16)	32.66 (6.57)	0.129	0.720	0.00 no effect
Risk-taking	35.92 (5.42)	36.84 (5.31)	3.818	0.051	0.05 no effect

*d: Cohen’s d*.

To examine the discriminative capacity of the BEPE in more depth, the sample was divided into three groups: employees, employees who plan to become self-employed in the next few months (potential entrepreneurs), and self-employed. This produced three groups of 931, 87, and 152 participants, respectively. The overall differences were statistically significant (*F*_16,2322_ = 3.676, *p* < 0.001, *d* = 0.32). In the Autonomy scale, the self-employed scored significantly higher than the employees group, in Innovation and Risk-taking, it was the potential entrepreneurs who were differentiated from the employees.

On the other hand, taking the total score in the META as a criterion, we selected 25% of subjects with lower scores and 25% of subjects with higher scores. Using a stepwise binary logistic regression, we inquired about which BEPE scales were capable of predicting belonging to these extreme groups. Three scales were selected: Innovativeness, Achievement motivation, Risk-taking (Nagelkerke’s *R*^2^ = 0.815 and percentage of correctly classified cases = 92.2%).

Finally, given that age can be a variable related to the *entrepreneurial spirit*, we have explored the relationship of the BEPE scales with age within each group of subjects (employees, potential entrepreneurs, self-employed) using stepwise regressions with age as a variable dependent and the BEPE scales as independent variables (See Table 8).

**Table 8:** Stepwise linear regressions by groups.

Groups	Standardized regression coefficients (β)						*R*^2^
Self-employed	–	–	–	–	–		
Employed	SE = –0.270	AU = 0.125	OP = 0.148	IN = –0.159	IL = –0.126	AM = 0.155	0.061
Potential entrepreneurs	RT = –0.408	AU = 0.344					0.138

The predictive capacity of the two types of personality traits (general vs. specific), represented by the Big-Five model and the BEPE scales, was put to the test via binary logistic regression. In both cases the predictive capacity was low, although it was improved when the NEO-FFI block of variables was added to the BEPE scales (Nagelkerke’s *R*^2^ went from 0.055 to 0.068, an increase of 1.3%). If we use the employees group and the potential entrepreneurs group as criteria, the predictive capacity is slightly higher and follows the same pattern (Nagelkerke’s *R*^2^ goes from 0.060 to 0.070, an increase of 1%).

## Discussion

Recent decades have seen a growing interest in the study of entrepreneurship from economic, social, and psychological perspectives. One of the main focuses of attention from a psychological perspective, in addition to cognitive factors, has been the study of entrepreneurs’ specific personality characteristics as opposed to the big personality traits described by models like the Big-Five. Despite this interest, there are not many measuring instruments designed to systematically and comprehensively evaluate these specific personality characteristics. This research addressed the adaptation of one of these tests to an adult population and the study of its psychometric properties. The test is the *Battery for the Assessment of the Enterprising Personality (BEPE)*, originally created to evaluate adolescents. The primary objective of this research was to gather evidence that the structure of the BEPE test (Muñiz et al., 2014; Suárez-Álvarez et al., 2014) for adolescents was conserved in the adult version. The results of the CFA show that the best fit model is the Bifactor with a general factor (entrepreneurship) and eight facets. The reliability coefficients are very high, both for the eight scales and overall. The internal convergent validity evidence is not completely satisfactory, but the discriminant validity was good. So, it seems reasonable to defend a Bifactor structure such as the CFA shows.

Another important part of this research was the search for evidence of convergent validity with external variables via the study of the relationship between the BEPE and the META, the most internationally well known scale for evaluating entrepreneurial personality from the point of view of specific traits The results show a good level of convergence whether we look at the correlational analysis or the discrimination between extreme groups. The correlation between the overall BEPE and META can be termed as good according to the criteria of the European Federation of Psychologists’ Associations Test Review Model (Evers et al., 2013). In terms of the relationship between the specific-trait perspective and the general-personality-trait perspective, the results of the canonical correlation analysis and the correlations between the scales indicate a moderate relationship between the two approaches.

The discriminative capacity of the BEPE scale between different groups, established *ad hoc* according to participants’ work status, was limited, although the trend was in the expected direction. These results about discriminating between groups must be taken with precaution, and we must wait for data from larger and representative groups of entrepreneurs. As Henrekson and Sanandaji (2014) indicated, the criterion for differentiating entrepreneurs from non-entrepreneurs is itself problematic. In this study we used being self-employed or not as the criterion for simplicity’s sake, while being aware that being self-employed is not synonymous with entrepreneurial spirit (Hurst and Pugsley, 2011). The results seem be in line with that idea, indicating that discriminative capacity between groups is higher when the “potential entrepreneurs” are included with the self-employed. The predictive capacity of specific traits, tested with the regression model, though low, was in line with other results indicating that specific traits would add predictive capacity to the general trait model (Leutner et al., 2014). When interpreting the predictive capacity of personality dimensions it should be remembered that personality factors are only a small part of the multiple individual, social, cultural and contextual factors which can potentially influence entrepreneurship, as is well indicated by those general models which aim to explain entrepreneurial spirit (Rauch and Frese, 2007a; Suárez-Álvarez and Pedrosa, 2016).

Regarding the age variable, the relationship found within the group of “potential entrepreneurs” is interesting and invites to investigate the role of age, together with other variables, as modulators of the “entrepreneurial spirit” (Bohlmann et al., 2017).

In conclusion, the version of BEPE for the adult population replicates and improves on the psychometric properties of the original version for young people, and exhibits very good evidence of convergent validity. It is, therefore, a measuring instrument that may be used in research and applied contexts. When interpreting the results, and when using the instrument itself, certain limitations of this research should be borne in mind. It was not possible to have clearly defined groups of entrepreneurs and non-entrepreneurs to assess the instrument’s discriminative capacity. It is precisely this limitation that provides the outline for future research to find validity evidence, which is already underway. Cross-cultural studies are also needed to evaluate entrepreneurial personality characteristics in different socio-cultural contexts (Byrne and van der Vijver, 2017). In order to carry out evaluations in applied contexts, a shorter form of the BEPE is needed, as is a computerized adaptation (Pedrosa et al., 2016; Nieto et al., 2017). The use of implicit measures to avoid the possible self-reports biases is another promising research line (Martínez-Loredo et al., 2018). Research into the entrepreneurial personality has only just begun, and the results are very promising, although there is a long road to follow. We believe that beginning with evaluation instruments is a good strategy, as they are the foundation that gives us to precise diagnoses, which in turn, will lead to effective interventions, the ultimate aim of all research in psychology.

## Ethics Statement

The study was not explicitly reviewed by an Ethics Committee, given that this is not required by our University of Oviedo, nor by the national guidelines established in the Code of Ethics of the Spanish Psychological Association. There are several reasons why an explicit approval by an Ethics Committee was not necessary: the participants evaluated were adults, the evaluation was voluntarily accepted, that is, an implicit informed consent is assumed, and the data is treated anonymously and confidentially. In addition, all the recommendations established in ISO-10667 Standard for the evaluation of people were strictly followed.

The whole evaluation process and the use of the measuring instruments were carried out always following the Deontological Code of the Spanish Psychological Association (2010), as well as the International Test Commission Guidelines for Test Use (2013).

Deontological Code of the Spanish Psychological Association (2010). Madrid: Consejo General de Colegios Oficiales de Psicólogos (www.cop.es).
International Test Commission Guidelines for Test Use (2013). International Test Commission: www.intestcom.org.

## Author Contributions

JM, JS-Á, and EG-C contributed conception and design of the study. LL organized the database. MC performed the statistical analysis and wrote the first draft of the manuscript. All authors contributed to manuscript revision, read and approved the submitted version. The views expressed in the paper represent the views of the individual authors and do not represent an official position of the Organisation for Economic Co-operation and Development.

## Conflict of Interest Statement

The authors declare that the research was conducted in the absence of any commercial or financial relationships that could be construed as a potential conflict of interest.

## References

[B1] Ahmetoglu G. Chamorro-Premuzic T. (2013). *META. Technical Manual.* London: Metaprofiling Ltd.

[B2] Ahmetoglu G. Leutner F. Chamorro-Premuzic T. (2011). EQ-nomics: Understanding the relationship between individual differences in trait emotional intelligence and entrepreneurship. *Pers. Individ. Differ.* 51 1028–1033. 10.1016/j.paid.2011.08.016

[B3] Almeida P. I. L. Ahmetoglu G. Chamorro-Premuzic T. (2014). Who wants to be an entrepreneur? The relationship between vocational interests and individual differences in entrepreneurship. *J. Career Assess.* 22 102–112. 10.1177/1069072713492923

[B4] American Educational Research Association [AERA], American Psychological Association [APA], and National Council of Measurement in Education [NCME] (2014). *Standards for Educational and Psychological Testing.* Washington, DC: AERA.

[B5] Baum J. R. Frese M. Baron R. A. Katz J. A. (2007). “Entrepreneurship as an area of psychology study: an introduction,” in *The Psychology of Entrepreneurship*, eds Baum J. R. Frese M. Baron R. A. (London: Lawrence Erlbaum), 1–18.

[B6] Betz N. E. Borgen F. H. Harmon L. W. (2005). *Manual for the Skills Confidence Inventory: Revised Edition.* Mountain View, CA: Consulting Psychologists Press.

[B7] Bohlmann C. Rauch A. Zacher H. (2017). A Lifespan perspective on entrepreneurship: Perceived opportunities and skills explain the negative association between age and entrepreneurial activity. *Front. Psychol.* 8:2015. 10.3389/fpsyg.2017.02015 29250004 PMC5717012

[B8] Brandstatter H. (2011). Personality aspects of entrepreneurship: a look at five meta-analysis. *Pers. Individ. Differ.* 51 222–230. 10.1016/j.paid.2010.07.007

[B9] Byrne B. M. van der Vijver F. J. R. (2017). The maximum likelihood alignment approach to testing for approximate measurement invariance: a paradigmatic cross-cultural application. *Psicothema* 29 539–551. 10.7334/psicothema2017.178 29048316

[B10] Caird S. (2006). “General measure of enterprising tendency version 2 (GET2),” in *Entrepreneurship and Innovation*, ed. Mazzarol T. (Prahran, VIC: Tilde University Press), 247–266.

[B11] Chandra Y. (2018). Mapping the evolution of entrepreneurship as a field of research (1990-2013): a scientometric analysis. *PLoS One* 13:e0190228. 10.1371/journal.pone.0190228 29300735 PMC5754054

[B12] Chell E. (2008). *The Entrepreneurial Personality: A Social Construction.* New York, NY: Routledge 10.4324/9780203938638

[B13] Chen Y.-H. Lin Y. J. (2018). Validation of the short self-regulation questionnaire for taiwanese college students (TSSRQ). *Front. Psychol.* 9:259. 10.3389/fpsyg.2018.00259 29551987 PMC5840206

[B14] Cheng F. F. Zang Z. (2018). “Bifactor models in psychometric test development,” in *The Wiley Handbook of Psychometric Testing. A Multidisciplinary Reference on Survey, Scale and Test Development*,eds Irwing P. Booth T. Hughes D. J. (Hoboken, NJ: Wiley-Blackwell).

[B15] Cordero A. Pamos A. Seisdedos N. (2008). *NEO PI-R, Inventario de Personalidad NEO Revisado. [Revised NEO Personality Inventory].* Madrid: TEA Ediciones.

[B16] Corp I. B. M. (2016). *IBM SPSS Statistics for Windows, Version 24.0.* Armonk, NY: IBM Corp.

[B17] Costa H. Ripoll P. Sánchez M. Carvalho C. (2013). Emotional intelligence and self-efficacy: effects on psychological well-being in college students. *Span. J. Psychol.* 16:E50. 10.1017/sjp.2013.39 23866247

[B18] Costa P. T. McCrae R. R. (1985). *The NEO Personality Inventory Manual.* Odessa, FL: Psychological assessment resources.

[B19] Cronbach L. J. (1951). Coefficient alpha and the internal structure of tests. *Psychometrika* 16 297–334. 10.1007/BF02310555

[B20] Damon W. Bronk K. C. Porter T. (2015). “Youth entrepreneurship,” in *Emerging Trends in the Social and Behavioral Sciences: An Interdisciplinary, Searchable, and Linkable Resource*, eds Scott R. A. Kosslyn S. M. (Hoboken, NJ: Wiley Online Library), 1–13. 10.1002/9781118900772.etrds0391

[B21] Di Fabio A. Bucci O. Gori A. (2016). High entrepreneurship, leadership, and professionalism (HELP): toward an integrated, empirically based perspective. *Front. Psychol.* 7:1842. 10.3389/fpsyg.2016.01842 27933015 PMC5122743

[B22] Evers A. Hagemeister C. Høstmælingen A. Lindley P. Muñiz J. Sjöberg A. (2013). *EFPA Review Model for the Description and Evaluation of Psychological Tests: Test Review form and Notes for Reviewers: Version 3.42.* Brussels: EFPA Standing Committee on Tests and Testing.

[B23] Favretto G. Pasini M. Sartori R. (2003). Attitudine imprenditoriale e misura psicométrica: il TAI. *Rev. Psicol. Lav. dellˊOrganizzazione* 9 271–282.

[B24] Ferrando P. J. Lorenzo-Seva U. (2017). Program factor at 10: origins, development and future directions. *Psicothema* 29 236–240. 10.7334/psicothema2016.304 28438248

[B25] Fornell C. Larcker D. F. (1981). Evaluating structural equation models with unobservable variables and measurement error. *J. Mark. Res.* 18 30–50. 10.2307/3151312

[B26] Global Entrepreneurship Monitor [GEM] (2018). *Global Report 2017/18.* London: London Business School.

[B27] Hair J. F. Black W. C. Babin B. J. Anderson R. E. (2009). *Multivariate Data Analysis*, 7th Edn Upper Saddle River, NJ: Prentice Hall.

[B28] Henrekson M. Sanandaji T. (2014). Small business activity does not measure entrepreneurship. *Proc. Natl. Acad. Sci. U.S.A.* 111 1760–1765. 10.1073/pnas.1307204111 24449873 PMC3918828

[B29] Horn J. L. (1965). A rationale and test for the number of factors in factor analysis. *Psychometrika* 30 179–185. 10.1007/BF02289447 14306381

[B30] Hu L. Bentler P. M. (1999). Cutoff criteria for fit indexes in covariance structure analysis: conventional criteria versus new alternatives. *Struct. Equ. Model.* 6 1–55. 10.1080/10705519909540118

[B31] Hurst E. Pugsley B. (2011). What do small businesses do? *Brookings Pap. Econ. Act.* 42 73–118. 10.1353/eca.2011.0017

[B32] Jackson D. L. Gillaspy J. A. Purc-Stephenson R. (2009). Reporting practices in confirmatory factor analysis: an overview and some recommendations. *Psychol. Methods* 14 6–23. 10.1037/a0014694 19271845

[B33] Kline R. B. (2011). *Principles and Practice of Structural Equation Modeling.* New York, NY: Guilford Press.

[B34] Lazarus R. S. Folkman S. (1984). *Stress, Appraisal and Coping.* New York, NY: Springer.

[B35] Leutner F. Ahmetoglu G. Akhtar R. Chamorro-Premuzic T. (2014). The relationship between the entrepreneurial personality and the Big Five personality traits. *Pers. Individ. Differ.* 63 58–63. 10.1016/j.paid.2014.01.042

[B36] Liñán F. Chen Y. W. (2006). *Testing the Entrepreneurial Intention Model on a Two-Country Sample.* Bellaterra: Universitat Autònoma de Barcelona.

[B37] Liñán F. Fayolle A. (2015). A systematic literatura review on entrepreneurial intentions: citation, thematic analysis and research agenda. *Int. Entrep. Manag. J.* 11 907–933. 10.1007/s11365-015-0356-5

[B38] Lorenzo-Seva U. Ferrando P. J. (2013). FACTOR 9.2 a comprehensive program for fitting exploratory and semiconfirmatory factor analysis and IRT models. *Appl. Psychol. Meas.* 37 497–498. 10.1177/0146621613487794

[B39] Lozano L. M. García-Cueto E. Muñiz J. (2008). Effect of the number of response categories on the reliability and validity of rating scales. *Methodology* 4 73–79. 10.1027/16142241.4.2.73 27403207

[B40] Marôco J. (2014). *Analise de Equacoes Estruturais: Fundamentos Teoricos, Software & Aplicacoes*, 2nd Edn. Lisbon: Pero Pinheiro.

[B41] Martínez-Loredo V. Cuesta M. Lozano L. M. Pedrosa I. Muñiz J. (2018). Multifactor implicit measures to assess enterprising personality dimensions. *Psicothema* 30 357–363. 10.7334/psicothema2018.204 30353834

[B42] McDonald R. P. (1999). *Test Theory: A Unified Treatment.* Mahwah: Lawrence Erlbaum Associates, Inc.

[B43] Moore S. Gullone E. (1996). Predicting adolescent risk behavior using a personalized cost benefit analysis. *J. Youth Adolesc.* 15 343–359. 10.1007/BF01537389

[B44] Muñiz J. Fidalgo A. M. García-Cueto E. Martínez R. Moreno R. (2005). *Análisis de Los Ítems [Item analysis].* Madrid: La Muralla.

[B45] Muñiz J. Suárez-Álvarez J. Pedrosa I. Fonseca-Pedrero E. García-Cueto E. (2014). Enterprising personality profile in youth: components and assessment. *Psicothema* 26 545–553. 10.7334/psicothema2014.182 25340904

[B46] Muthén L. K. Muthén B. O. (2017). *Mplus User’s Guide*, 8th Edn. Los Angeles, CA: Muthén & Muthén.

[B47] Nieto M. D. Abad F. J. Hernández-Camacho A. Garrido L. E. Barrada J. R. Aguado D. (2017). Calibrating a new item pool to adaptively assess the Big Five. *Psicothema* 29 390–395. 10.7334/psicothema2016.391 28693712

[B48] Obschanka O. Hakkarainen K. Lonka K. Salmeda-Aru K. (2017). Entrepreneurship as a twenty-first century skill: entrepreneurial alertness and intention in the transition to adulthood. *Small Bus. Econ.* 48 487–501. 10.1007/s11187-016-9798-6

[B49] OECD (2017). *Entrepreneurship at a Glance 2017.* Paris: OECD Publishing.

[B50] OECD/The European Commission (2013). *The Missing Entrepreneurs: Policies for Inclusive Entrepreneurship in Europe.* Brussels: OECD Publishing 10.1787/9789264188167-en

[B51] Pedrosa I. Suárez-Álvarez J. García-Cueto E. Muñiz J. (2016). A computerized adaptative test for enterprising personality assessment in youth. *Psicothema* 28 471–478. 27776618 10.7334/psicothema2016.68

[B52] Rauch A. Frese M. (2007a). “Born to be an entrepreneur? Revisiting the personality approach to entrepreneurship,” in *The Psychology of Entrepreneurship*, eds Baum J. R. Frese M. Baron R. J. (Mahwah, NJ: Erlbaum), 41–65.

[B53] Rauch A. Frese M. (2007b). Let’s put the person back into entrepreneurship research: a meta-analysis on the relationship between business owners’ personality traits, business creation, and success. *Eur. J. Work Organ. Psychol.* 16 353–385. 10.1080/13594320701595438

[B54] Reise P. S. (2012). The rediscovery of bifactor measurement models. *Multivariate Behav. Res.* 47 667–696. 10.1080/00273171.2012.715555 24049214 PMC3773879

[B55] Reise P. S. Bonifay W. Haviland M. G. (2018). “Bifactor modelling and the evaluation of scale scores,” in *The Wiley Handbook of Psychometric Testing. A Multidisciplinary Reference on Survey, Scale and Test Development*, eds Irwing P. Booth T. Hughes D. J. (Hoboken, NJ: Wiley-Blackwell).

[B56] Sánchez J. C. (2010). Evaluación de la personalidad emprendedora: Validez factorial del cuestionario de orientación emprendedora (COE). *Rev. Latinoam. Psicol.* 42 41–52.

[B57] Sánchez J. C. (2011). Entrepreneurship as a legitimate field of knowledge. *Psicothema* 23 433–438. 21774896

[B58] Shepperd J. A. Carroll P. Grace J. Terry M. (2002). Exploring the causes of comparative optimism. *Psychol. Belg.* 42 65–98. 1301783

[B59] Sinval J. Marques-Pinto A. Queirós C. Marôco J. (2018). Work engagement among rescue workers: Psychometric properties of the Portuguese UWES. *Front. Psychol.* 8:2229. 10.3389/fpsyg.2017.02229 29403403 PMC5786829

[B60] Suárez-Álvarez J. Campillo-Álvarez A. Fonseca-Pedrero E. García-Cueto E. Muñiz J. (2013). Professional training in the workplace: The role of achievement motivation and locus of control. *Span. J. Psychol.* 16:E35. 10.1017/sjp.2013.19 23866230

[B61] Suárez-Álvarez J. Pedrosa I. García-Cueto E. Muñiz J. (2014). Screening enterprising personality in youth: an empirical model. *Span. J. Psychol.* 17:E60. 10.1017/sip.2014.61 26055899

[B62] Suárez-Álvarez J. Pedrosa J. (2016). Evaluación de la personalidad emprendedora: situación actual y líneas de futuro. *Papeles Psicólogo* 37 62–68.

[B63] van der Linden W. (ed.) (2016). *Handbook of Item Response Theory*, Vol. 3 Boca Ratón, FL: Chapman & Hall/CRC, 87–103

[B64] Van Gelderen M. Jansen P. (2006). Autonomy as a start-up motive. *J. Small Bus. Enterp. Dev.* 13 23–32. 10.1108/14626000610645289

[B65] Wang J. Wang X. (2012). *Structural Equation Modeling; Aplications Using Mplus.* Hoboken, NJ: John Wiley & Sons 10.1002/9781118356258

[B66] Zhao H. Seibert S. E. Lumpkin G. T. (2010). The relationship of personality to entrepreneurial intentions and performance: a metaanalytic review. *J. Manag.* 36 381–404. 10.1177/0149206309335187

