# Corrigendum: Assessment of Eight Entrepreneurial Personality Dimensions: Validity Evidence of the BEPE Battery

**DOI:** 10.3389/fpsyg.2019.00725

**Published:** 2019-04-09

**Authors:** Marcelino Cuesta, Javier Suárez-Álvarez, Luis M. Lozano, Eduardo García-Cueto, José Muñiz

**Affiliations:** ^1^CIBERSAM, Department of Psychology, University of Oviedo, Oviedo, Spain; ^2^Directorate for Education and Skills, Organisation for Economic Co-operation and Development, Paris, France; ^3^Mind, Brain and Behavior Research Center, Department of Methodology of Behavioral Sciences, University of Granada, Granada, Spain

**Keywords:** personality, entrepreneurs, assessment, validity evidence, Big Five

In the original article, there was a mistake in Table 2 as published. Several negative signs in the Item column are missing. The corrected Table 2 appears below.

**Table 2:** Factor loadings of Bifactor Model.

**Item**	**Factor loading**	**Item**	**Factor loading**	**Item**	**Factor loading**	**Item**	**Factor loading**
**General factor**							
SE1	0.72	IN1	0.48	AM1	0.59	ST1	0.72
SE2	0.75	IN2	0.62	AM2	0.67	ST2	0.27
SE3	0.71	IN3	0.56	AM3	0.61	ST3	0.54
SE4	0.69	IN4	0.63	AM4	0.52	ST4	0.37
SE5	0.70	IN5	0.48	AM5	0.67	ST5	0.46
SE6	0.79	IN6	0.56	AM6	0.70	ST6	0.42
SE7	0.65	IN7	0.60	AM7	0.60	ST7	0.38
SE8	0.72	IN8	0.74	AM8	0.69	ST8	0.42
SE9	0.78	IN9	0.58	AM9	0.66	ST9	0.36
SE10	0.70	IN10	0.56	AM10	0.58	ST10	0.53
AU1	0.43	IL1	0.47	OP1	0.59	RT1	0.61
AU2	0.14	IL2	0.63	OP2	0.55	RT2	0.73
AU3	0.61	IL3	0.50	OP3	0.56	RT3	0.50
AU4	0.63	IL4	0.52	OP4	0.62	RT4	0.56
AU5	0.32	IL5	0.42	OP5	0.79	RT5	0.66
AU6	0.26	IL6	0.31	OP6	0.62	RT6	0.59
AU7	0.30	IL7	0.60	OP7	0.50	RT7	0.57
AU8	0.52	IL8	0.39	OP8	0.51	RT8	0.64
AU9	0.55	IL9	0.43	OP9	0.71	RT9	0.56
AU10	0.56	IL10	0.46	OP10	0.48	RT10	0.46
**SE**	**AU**	**IN**	**IL**	**AM**	**OP**	**ST**	**RT**
**Group factors**							
−0.02	0.47	0.48	0.69	0.40	0.57	0.20	0.39
−0.28	0.61	0.49	0.40	0.37	0.56	0.47	0.28
0.54	0.18	0.30	0.61	0.34	0.39	0.41	0.55
−0.15	0.02	0.45	0.30	0.37	0.45	0.42	0.19
0.24	0.70	0.32	0.44	−0.07	0.31	0.48	0.21
−0.02	0.66	0.49	0.43	0.05	0.44	0.74	0.60
0.49	0.67	0.49	0.24	0.26	0.58	0.67	0.14
0.12	0.52	0.35	0.64	0.06	0.47	0.47	0.28
0.11	0.36	0.41	0.55	0.46	0.19	0.50	0.18
−0.26	0.21	0.41	0.66	0.58	0.61	0.23	0.50

*SE, self-efficacy; AU, autonomy; IN, innovativeness; IL, internal locus of control; AM, achievement motivation; OP, optimism; ST, stress tolerance; RT, risk-taking*.

The authors apologize for this error and state that this does not change the scientific conclusions of the article in any way. The original article has been updated.

